# Affective state estimation based on Russell’s model and physiological measurements

**DOI:** 10.1038/s41598-023-36915-6

**Published:** 2023-06-16

**Authors:** Roberto Cittadini, Christian Tamantini, Francesco Scotto di Luzio, Clemente Lauretti, Loredana Zollo, Francesca Cordella

**Affiliations:** Research Unit of Advanced Robotics and Human-Centred Technologies, Department of Engineering, Università Campus Bio-Medico di Roma, Rome, Italy

**Keywords:** Biomedical engineering, Health care, Emotion

## Abstract

Affective states are psycho-physiological constructs connecting mental and physiological processes. They can be represented in terms of arousal and valence according to the Russel’s model and can be extracted from physiological changes in human body. However, a well-established optimal feature set and a classification method effective in terms of accuracy and estimation time are not present in the literature. This paper aims at defining a reliable and efficient approach for real-time affective state estimation. To obtain this, the optimal physiological feature set and the most effective machine learning algorithm, to cope with binary as well as multi-class classification problems, were identified. ReliefF feature selection algorithm was implemented to define a reduced optimal feature set. Supervised learning algorithms, such as K-Nearest Neighbors (KNN), cubic and gaussian Support Vector Machine, and Linear Discriminant Analysis, were implemented to compare their effectiveness in affective state estimation. The developed approach was tested on physiological signals acquired on 20 healthy volunteers during the administration of images, belonging to the International Affective Picture System, conceived for inducing different affective states. ReliefF algorithm reduced the number of physiological features from 23 to 13. The performances of machine learning algorithms were compared and the experimental results showed that both accuracy and estimation time benefited from the optimal feature set use. Furthermore, the KNN algorithm resulted to be the most suitable for affective state estimation. The results of the assessment of arousal and valence states on 20 participants indicate that KNN classifier, adopted with the 13 identified optimal features, is the most effective approach for real-time affective state estimation.

## Introduction

Affective state changes in a person are always accompanied by significant physiological responses in human organs and tissues such as brain, heart, skin, blood flow, muscles and sometimes also facial expressions and voice^1,2^. According to Russell’s model^3^, each affective state can be represented by two dimensions: arousal and valence. Arousal indicates the level of a person’s involvement in reaction to a stimulus. Anger induces an intense physiological response, unlike other slighter states such as boredom that do not provide the same physiological alteration. Valence defines the positive or negative state in response to a stimulus. High values of valence characterize pleasant situations, meanwhile low values are attributed to unpleasant ones that can induce states of stress, anxiety, or irritation.

Objective methods for the assessment of the person’s affective state could be relevant in different application fields, such as: (1) robot-aided rehabilitation^4^, to adapt the behavior of a robotic system according to the patient’s state during the rehabilitation therapy. This could be useful for providing only the necessary assistance to the patient considering engagement and effort during the rehabilitation and for allowing him/her to exploit his/her residual abilities; (2) in assistance^5^ and prosthetic fields^6,7^, to evaluate the patient’s acceptability of a device to be used for the rest of his/her life; (3) in the corporate field^8^, to assess particularly stressful periods that can adversely affect the wellness of the employees and consequently burden on the productivity of the entire company; (4) in the automotive field, to reduce the number of road crashes. In fact, stress and anger negatively affect the driving task^9^. The assessment of the driver’s affective state, as well as for airplane pilot of intercontinental flights^10^, is a considerable help for his/her and others’ safety. However, real-world settings introduce many challenges for emotion recognition tasks such as the lack of ground-truth labels, context dependency, uncontrolled subject movements affecting the measurements and sensors placement^11^. Given the undisputed usefulness of an affective state estimation model to be used in real world applications, experiments conducted in highly structured environments are needed to explore the possibility to use wearable instrumentation to estimate the user state.

Time needed for affective state estimation is as important as the accuracy of the estimation itself^12^. An affective state recognition system must be able to provide reliable real-time output in order to contribute with a sensible and effective help in the aforementioned fields of application. Real-time systems are governed by a dual concept of correctness: logical (the system produces the expected result) and temporal (the result is produced over time). Such systems must meet stringent time constraints (deadlines) as they must continuously interact with the surrounding environment to control, manage or report significant events within a predetermined time. Therefore, the entire response cycle must occur within a certain period T, which is characteristic of the physics of the system to be controlled (human affective state). Changes in the human affective state are of the order of seconds. In the aforementioned application scenarios, fast timing and high prediction accuracy are required, as real-time information on patients/workers is needed to estimate their affective state and identify possible risk situations and adapt technologies to human behavior. The estimation of the affective state is a valuable assessment that can be exploited to better understand the user’s state in human-centered technologies, as long as it is provided in due time and with good accuracy.

Studies about emotion recognition from physiological signal acquisition, differing among each other for several aspects, are present in the literature: (1) emotional model selected for the description of the evoked affective states. One category of models focuses on the valence-arousal plane to describe user emotional experience^13^, meanwhile the second category of models argues for the possibility of labeling the emotional experience in discrete basic emotions^14^; (2) materials of stimulation such as images, sounds, videos to evoke specific affective states^15^. These stimuli could be scientifically validated or arbitrarily chosen by experimenters; (3) physiological signals acquired: reliable association exists between the activation of the Autonomic Nervous System (ANS) and the affective response of physiological signals such as sweating, cardiac and breathing activity changes^16^. These physiological processes have been investigated in previous works in the literature through the partial or total combination of skin conductance, cardiac and respiratory activity: galvanic skin response and heart activity are coupled for discrete emotion recognition using Artificial Neural Network^17^ and for high-low valence and arousal level classification using Support Vector Machine and K-Nearest Neighbors classifiers^18^ with 14 and 21 features, respectively; respiration and cardiac activity are employed for discrete emotion recognition using statistical significant differences for the spectral and temporal domains^19^; Heart rate variability is used with the Support Vector Machine for arousal (accuracy 48% with 6 features) and valence classification (accuracy 42% with 2 features), recommending the integration of skin activity and respiration^20^. These are valuable biometric signals allowing to reveal information about unconscious behaviors that are not under cognitive control. Indeed, they cannot be consciously controlled, falsified or kept hidden by the person during the experimentation^21^. In particular, the changes in the skin electrical properties provide information about the arousal level of a person^22^ as well as on the cognitive load^23^. Cardiac and respiratory activities are also excellent markers for estimating changes in mental state: heart and respiration rates manifest an increase or decrease with respect to the rest condition, according to the stimulus presented to the person. Such physiological metrics are considered the most suitable for assessing changes in human affective state in the aforementioned application fields, both in terms of reliability and obtrusiveness for the subject. Affective state estimation should not rely on obtrusive sensors as they should not compromise and/or hinder the patient’s rehabilitation tasks or the worker’s daily activities. Some works add features coming from facial expressions, gestures, or speech to physiological signals with the aim of improving affective recognition^24–26^. However, facial, speech or gesture recognition are not always suitable to estimate affective state, in particular for post-stroke patients, severe psychiatric patients or small children, since they are unable to express them properly; (4) machine learning algorithms used for affective state estimation^1^. The most simple machine learning classifier that can be implemented to estimate the user’s affective state is the Linear Discriminant Analysis (LDA). This linear classifier assigns the prediction by projecting the feature values to a new subspace^27^. Non-linear approaches, such as the Support Vector Machines (SVM) and the K-Nearest Neighbors (KNN), calculate the decision boundary accurately outperforming the linear ones, particularly when using physiological parameters as inputs. Moreover, different SVM kernels, such as the cubic (SVMc) and the gaussian (SVMg) ones, are worth to be tested in order to improve the classification accuracy in affective state estimation^28^. At least, KNN algorithm proved to be a suitable solution to effectively recognize the participants’ affective state evoked by visual stimuli^29^.

The literature analysis does not suggest an established feature set that constitutes the optimal one to be used for person’s affective state estimation: some feature selection approaches exclude non-relevant features and others transform the original ones into a new feature set.

Furthermore, it is evident that a lack of comparison between the performance, in terms of accuracy and estimation time, of the well-known machine learning algorithms adopted both for simple binary cases and for complex multi-class ones.

This paper addresses these literature lacks and aims at proposing the optimal feature set selection for real-time affective state estimation and a comparison between the machine learning algorithms most employed in the psychophysiological field (i.e. KNN, SVMc, SVMg, and LDA) establishing which machine learning algorithm could be the more accurate and responsive for real-time affective state estimation.

Twenty healthy volunteers have been involved to validate the proposed approach and the valence-arousal based emotional model was chosen to classify participants’ affective states. Existing physiological databases in literature are built with data collected while watching multimedia contents selected by experimenters, not part of publicly available scientifically validated databases^30,31^. The stimulation material used in this work consists of visual stimuli taken from a scientifically validated images database^32^. Physiological signals taken into consideration are related to skin, cardiac and respiratory activities. In particular, for the purposes of this work: (1) a Physiological Monitoring Module has been developed to record and acquire biometric signals, (2) a Graphical User Interface (GUI) has been designed to administer visual stimuli during the experiment, (3) pre-processing and feature extraction have been conducted on the physiological raw data recorded, (4) a feature selection algorithm has been implemented to choose the most important predictors and to define the optimal feature set for improving classification accuracy, (5) both binary and multi-class classification problems have been addressed by training and testing different machine learning algorithms and evaluating them in terms of accuracy and estimation time.

## Materials and methods

**Figure 1:**
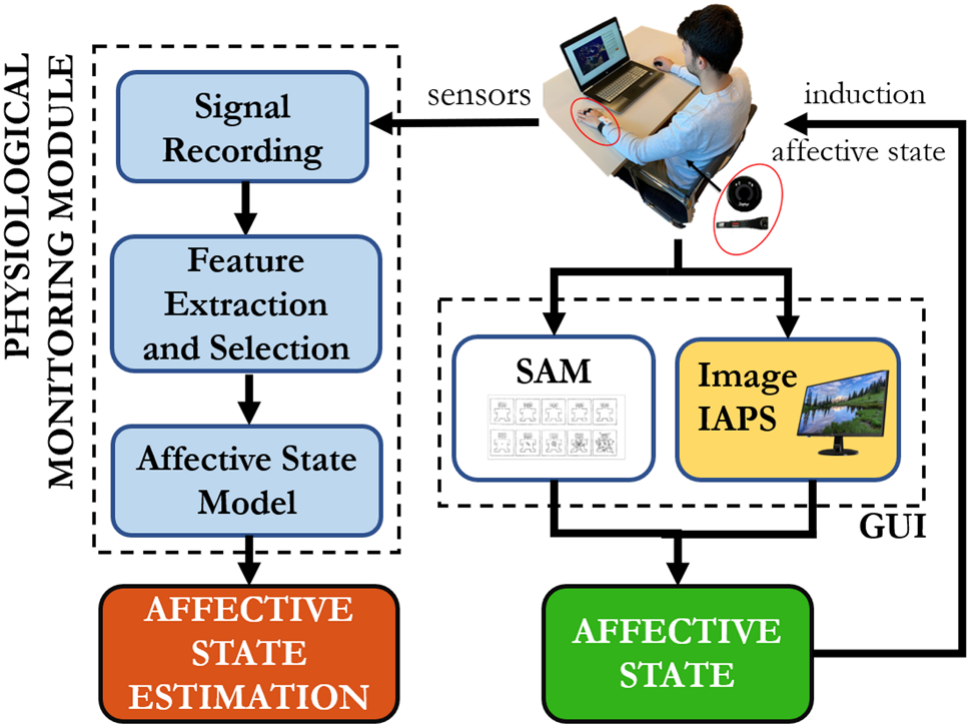
Main modules of the experimental workflow: Graphical User Interface (GUI) and Physiological Monitoring Module.

The basic building blocks of the proposed approach are shown in Fig. 1. Each participant wears sensors for measuring physiological parameters during his/her interaction with the GUI in the experimental scenario.

The Physiological Monitoring Module acquires physiological signals coming from wearable sensors (Signal Recording), processes them extracting and selecting relevant features (Feature Extraction and Selection) to be employed in different well-known machine learning algorithms in order to estimate participant’s affective state (Affective State Model). The GUI is constituted of two main blocks: the first one (IAPS Image) delivers visual stimuli to elicit affective states in the participant and the second one permits the participants to assess, through the module Self-Assessment Manikin (SAM)^33^, what the stimulus elicited in them. The GUI is developed in Microsoft Visual Studio 2019 using C# programming language.

### Machine learning classifiers

In this work, well-known machine learning algorithms already established in the literature have been implemented: (1) K-Nearest Neighbors (KNN), in which the predicted class following a new observation corresponds to the label of its K neighbouring elements; (2) cubic Support Vector Machine (SVMc) and gaussian Support Vector Machine (SVMg). Kernel method utilizes existing features, applies some transformations, and creates new features that are the key for SVM to find the nonlinear decision boundary; (3) Linear Discriminant Analysis (LDA), which aims at identifying a hyperplane that separates the elements of different classes.

The performance of each machine learning algorithm is evaluated by considering:

- *Accuracy*, the ratio between the sum of elements on the main diagonal of the confusion matrix and the elements of the whole confusion matrix;
- *F-score*, the harmonic average between precision, i.e. the fraction of relevant instances among the total retrieved instances, and recall, i.e. the fraction of relevant instances retrieved over the total amount of relevant instances. It is sufficient that only one of these two parameters is numerically low to drastically lower the value of F-score. In this way, if F-score is high it is sure that both precision and recall have high values;
- *Estimation time*, the time needed to the model for assigning the affective state. During this time span, the model receives physiological raw data in input, processes the signals with filtering, extracts all the features, selects them and provides affective state estimation.

### Affective states

**Figure 2:**
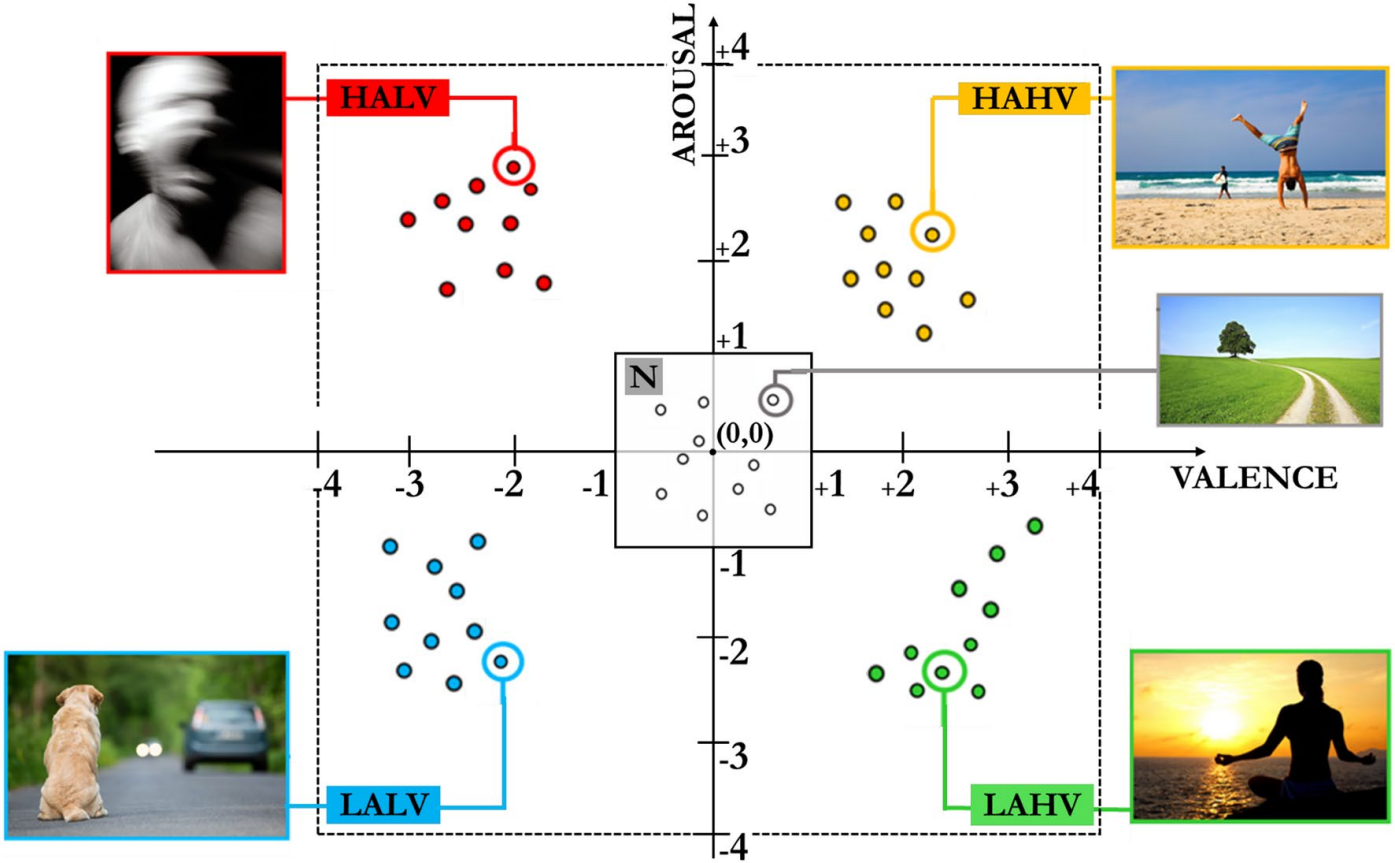
Valence-arousal plane divided into 5 quadrants: HAHV, HALV, LALV, LAHV, and N. The neutral region N is located in the center of the valence-arousal plane. The colored dots represent the location of the 50 IAPS images considered in this work.

In the valence-arousal plane, affective states are distinct from each other in different quadrants according to the value of the two dimensions of valence and arousal. In this work, scientifically validated visual stimuli are employed to elicit a real specific affective state in the participant: International Affective Picture System database (IAPS) is developed and kindly provided on request by the National Institute of Mental Health Center for Emotion and Attention of the University of Florida^32^ and includes 956 color pictures ranging from simple contents of ordinary life, e.g. home furniture or landscapes, to extremely rare scenes reporting violence or repellents capable of eliciting strong reactions during their vision. Each IAPS image is identified by a numerical evaluation of the affective state evoked in the observer, in terms of arousal and valence: both dimensions vary from a minimum value of 1 to a maximum value of 9, thus defining a 2D point belonging to one of the quadrants of the valence-arousal plane. In this work, these ratings were rescaled within a range of − 4 to 4. This processing was implemented to align the neutral region at the center point, which is denoted by the origin coordinates (0,0). According to this rescaled IAPS image’s numerical evaluation, 50 IAPS images are carefully selected from the database to evoke distinct affective states divided into the 5 quadrants of the valence-arousal plane, as shown in Fig. 2:

- High-Arousal-High-Valence quadrant (HAHV): positive and involved state of the person. Pleasure, joy, and excitement states belong to this quadrant;
- High-Arousal-Low-Valence quadrant (HALV): negative and involved state of the person. Anger, anxiety and fear states belong to this quadrant;
- Low-Arousal-Low-Valence quadrant (LALV): negative and uninvolved state of the person. Boredom and sleep states belong to this quadrant;
- Low-Arousal-High-Valence quadrant (LAHV): positive and uninvolved state of the person. Calm, relaxed, peaceful states belong to this quadrant;
- Neutral quadrant (N): affective states that cannot be unambiguously located in any of the aforementioned four quadrants (HAHV, HALV, LALV, and LAHL). The size of the neutral region is set at ±1 in both the valence and arousal axes, i.e. the 6.25% of the entire valence-arousal plane, around the origin coordinate (0, 0) of the affective space model, which is in alignment with the neutral region definition in previous works in the literature^34–36^. By employing this rescaled range, the proposed approach aims at creating a standardized framework where the origin accurately represents a state of neutrality or absence of emotional intensity. The neutral discrete affective state is more efficiently mapped by a cluster of points with the center in the origin coordinates (0, 0), rather than by a single unique point^37^.

In this way, one affective state evoked are related to one specific quadrant of the valence-arousal plane. Each quadrant of the valence-arousal plane represents a class for supervised learning adopting machine learning algorithms.

Affective state estimation is performed for both binary and multi-class problems. This is to compare machine learning algorithms in terms of accuracy and estimation time on different classification problems and to evaluate which approach has a greater impact on the identification of the affective state in the valence-arousal plane:

- 2 classes: high/low valence binary problem to identify positive (HAHV and LAHV quadrants) or negative (HALV and LALV quadrants) volunteer’s state;
- 2 classes: high/low arousal binary problem to identify involved (HAHV and HALV quadrants) or uninvolved (LALV and LAHV quadrants) volunteer;
- 4 classes: multi-class problem for identification of the 4 quadrants of Russell’s model (HAHV, HALV, LALV and LAHV quadrants);
- 5 classes: multi-class problem for identification of a fifth central quadrant representative of a neutral state (N) in addition to the 4 quadrants of Russell’s model.

### Physiological Monitoring Module

The Physiological Monitoring Module is implemented in MATLAB^®^ to transpose the physiological signals, synchronously acquired with the visual stimuli provided by the IAPS images, into an affective state estimation of the participant, after feature extraction and selection processes.

#### Signal recording

Physiological signals and parameters of interest in this work are Galvanic Skin Response (GSR), related to electrodermal skin activity and Heart Rate (HR), Heart Rate Variability (HRV), Respiration Rate (RR) related to cardiac and respiratory activities. These biometric signals are selected since they are regulated by the Autonomic Nervous System and therefore cannot be falsified or altered by the person during experimentation (ANS-controlled). The GSR raw signal is collected with a sampling frequency of 51.2 Hz and sent via Bluetooth 2.0. Electrocardiogram (ECG) and RR, acquired at 250 Hz at 25 Hz respectively, are sent via Bluetooth Low Energy (BLE) communication protocol. A Computer with Windows 10 Operating System equipped with Intel^®^ Core^™^ i7-7700HQ processor at 4 $\times$ 3.8 GHz, 16 GB of RAM, receives data from both the wearable devices and all the collected information is synchronized and saved under Yet Another Robot Platform (YARP)^38^ at 36.5 Hz.

#### Feature extraction and selection

A time window of 0.5 s is used to pre-process physiological data and extract features. The GSR raw signal is filtered with a low-pass Butterworth filter with a cutoff frequency of 5 Hz, to remove noise or other artifacts, and then decomposed in its tonic and phasic components. To retrieve the tonic level from GSR signal, also called Skin Conductance Level (SCL), a low-pass Butterworth filter is implemented with a cut-off frequency of 0.1 Hz. On the other hand, the Skin Conductance Response (SCR), that is the phasic response, can be extracted by filtering with a high-pass Butterworth filter with a cutoff frequency of 0.1 Hz^39^. Starting from SCL and SCR, 15 features are extracted: statistical metrics like the mean value (SCL_mean and SCR_mean), the standard deviation (SCL_std and SCR_std), the minimum (SCL_min and SCR_min), the maximum (SCL_max and SCR_max), linear combination of these ((SCL_max-SCL_min) and (SCR_max-SCR_min)), mean value of the first and second derivative of the SCL (respectively SCL_dot and SCL_ddot) and the number and amplitude of the SCR signal peaks. In the literature, a peak is defined as an increase in the SCR signal greater than 0.01–0.05 $\upmu$s^40^. In this particular application, the threshold for the peak detection algorithm is set to 0.03 $\upmu$s, exactly in the middle of the literature’s range. The number of peaks (N_peaks), the mean peaks amplitude (PA_mean) and its standard deviation (PA_std) are computed and used as features.

The ECG is analyzed to compute the inter-beat interval (IBI) and the instant HR expressed in beats per minute (bpm). The same statistical features introduced above are computed for the HR. In addition, two time domain metrics of the HRV are extracted^41^: the standard deviation of time interval between two successive normal heartbeats (sDNN) and the root mean square of successive heartbeats (rMSSD). Frequency domain HRV metrics cannot be used as features for real-time classification purposes because a time window of at least 2–5 min is required for a correct computation of the Fast Fourier Transform of the IBI signal^42^. This is not compatible with the proposed application, since the time window is 0.5 s. Anyway, 7 features related to cardiac activity are extracted.

The RR provides only the mean value collected for each time window of 0.5 s. The RR does not vary in such a small time, so that the standard deviation does not bring additional information.

Table 1 reports all the 23 extracted features from the collected physiological signals in the time window.

**Table 1:** Extracted features from the physiological measurement recorded in the experiment.

Physiological measure	Extracted features
GSR	SCL_mean, SCL_std, SCL_min, SCL_max, (SCL_max-SCL_min), SCL_dot, SCL_ddot, SCR_mean, SCR_std, SCR_min, SCR_max, (SCR_max-SCR_min), N_peaks, PA_mean, PA_std
ECG	HR_mean, HR_std, HR_min, HR_max, (HR_max-HR_min), sDNN_mean, rMSSD_mean
Respiration	RR_mean

Feature selection can be used to identify and remove unneeded, irrelevant, and redundant features from data that do not contribute to improve the accuracy of a predictive model or may even decrease the accuracy of the model itself.

Assessing the relevance of each computed feature per participant highlights which are the features assuming a greater impact on the classification problem with respect to others for the specific subject. Analyzing the outcomes among several participants allows identifying a globally optimal feature set. In this work, ReliefF algorithm is implemented^43^ to remove the least significant features for affective state estimation. ReliefF algorithm is adopted since it is indicated for multi-label feature selection^44^ and it can deal with multi-class problems without diminishing the quality of the classifiers constructed using the features selected^45^. It selects randomly an instance R_i_, then searches for k of its nearest neighbors from the same class, called nearest hits H_j_, and also k nearest neighbors from each of the different classes, called nearest misses M_j_. It updates the quality estimation for all attributes depending on their values for R_i_, hits H_j_ and misses M_j_ and assigns a weight W to each feature. These weights represent feature relevance in the classification problem. The parameter k can be properly tuned for any individual problem. In our study, the ReliefF algorithm is implemented for k = 1, 3, 5, 10 in order to assess how features’ weights is affected by the k parameter value. A global optimal feature set is obtained from all the starting features. Starting from the entire dataset of physiological features, ReliefF algorithm for feature selection is applied to the training set of each volunteer. In particular, the features returning a median positive weight among all the enrolled participants define the global optimal feature set.

#### Affective state estimation

Supervised learning allows to instruct a model through training data, consisting of pairs of inputs (features from physiological signals) and outputs (affective state classes). After successful training session, the model is able to elaborate predictions on the problem outputs from new inputs^46^. In this way, new general data as new input are classified as an affective state estimation output by the obtained model with a percentage of accuracy according to the machine learning algorithm chosen.

Regardless of the considered problem, i.e. binary or multi-class, machine learning algorithms are trained with physiological signals coming from the vision of the IAPS images that are related to affective states evoked. For each session of training, physiological signals related to one IAPS image for each class are voluntarily left out in order to employ them as new data never shown in input to the machine learning algorithm for testing. Moreover, participants express subjective self-assessment of their affective state, in terms of arousal and valence, using GUI during the experimental session. If a volunteer’s self-assessment is completely inconsistent with the assessment indicated in the IAPS database, the related physiological data are not considered so as not to adversely affect model training. First, training and testing are performed by machine learning algorithms taking into account all the 23 starting features. Then, training and testing are performed only with the most relevant predictive features extracted by the ReliefF algorithm, which identifies the optimal feature set through a ranking of weights.

#### Statistical analysis

Some statistical analysis is conducted on the resulting data. In particular, the variance analysis ANOVA is conducted to assess whether the introduction of the ReliefF algorithm improved significantly the classification accuracy. In addition, for each classification problem, multiple ANOVA comparisons are carried out in order to analyze if the accuracies of the implemented models are significantly different. Since multiple comparisons are carried out (i.e. KNN vs SVMc, KNN vs SVMg, KNN vs LDA, SVMc vs SVMg, SVMc vs LDA, and SVMg vs LDA), the *p* value is corrected by using the Bonferroni method^47^: $p_m = p/n_c$, where $p_m$ is the new value used for multiple comparisons, $p = 0.05$ is the original designated threshold of significance and $n_c=6$ is the number of multiple comparisons. Any of the 6 comparison is statistically significant if the resulting *p* value is $\le p_m = 0.008$.

### Experimental protocol and setup

In this study, 20 healthy volunteers (10 males, 10 females, 25 ± 2.3 years old) with no previous experience with the IAPS database, were enrolled. The volunteers are welcomed into the lab and introduced to the experiment. The study was approved by the Universitá Campus Bio-Medico di Roma Ethics Committee (Ethical Approval N. 03/19 PAR ComEt CBM) and in accordance with the Declaration of Helsinki. All participants have been adequately informed about the purpose of the study and gave their written informed consent. All the acquisitions take place inside an empty room, under similar lighting and acoustic conditions. The volunteer sits in front of a table, a 20-in. screen monitor showing the developed GUI and a mouse allowing him/her to interact with it. An experimenter assists during the entire acquisition session to ensure the correct recording of the physiological signals. The participant wears a chest strap belt for measuring cardiac and respiratory activities (i.e. Zephyr BioHarness 3.0) and a device for measuring electrodermal skin activity by positioning two electrodes on the proximal phalanges of the index and middle fingers (i.e. Shimmer 3 GSR+ Unit). It can be comfortably fixed on the wrist thanks to an elastic band. The volunteer sits on the chair letting his/her left arm lay comfortably on the table to allow the correct recording of the electrodermal skin signal. In this way, motion artifacts can be limited. The right hand of the participant is on the mouse interacting with the GUI. The GUI displays visual stimuli during the experiment to elicit the corresponding affective state in the participant and obtain his or her self-assessment. The SAM is administered via the GUI: each participant evaluates his or her affective state and selects an integer from 1 to 9 for both valence and arousal, moving the GUI track-bars with the cursor to indicate his or her sensation towards the displayed IAPS image. The GUI is designed to be easy to understand, intuitive for the user, and to make the experimental experience as immersive as possible. Furthermore, during the first part of the experiment, the GUI consists of an introductory phase that informs the participant about the experiment, instructs him/her on the SAM to correctly interpret the meanings of arousal and valence and shows him/her the tutorial on how to interact with the GUI itself. After this preliminary step to instruct the participant about the experiment, a physiological signals recording starts and lasts about 4 min. During this time, the volunteer is in his/her physiological rest condition, without any cognitive or physical task to perform. This step is essential because the baseline of physiological signals allows the normalization procedure, which consists in subtracting this baseline value from the current physiological signals recorded^46^. This pre-processing step is paramount to reduce the intrinsic intra and inter-subject variability of the physiological parameters raised from age, gender, time of day, sensors placement and other factors.

**Figure 3:**
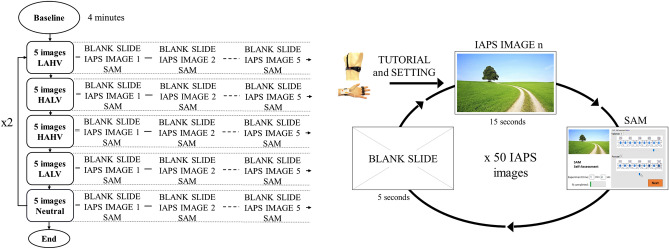
Experimental protocol: order of administration of visual stimuli by the GUI, organized in 10 different clusters of 5 IAPS images each.

As shown in Fig. 3, each volunteer receives 50 different visual stimuli organized in clusters of images belonging to the same specific quadrant. Then, 10 different clusters are organized into 5 IAPS images each. The clusters are presented in the following order twice for each participant: LAHV, HALV, HAHV, LALV, and N.

Regardless of the class of belonging, each IAPS image is presented to the volunteer for 15 s and the physiological measurements are recorded and analyzed^48^. Before displaying each IAPS image as a visual stimulus, a blank slide is shown to the participants for 5 s in order to restore the baseline physiological condition. After displaying each IAPS image, SAM scale is shown to the participants to allow them to provide the self-assessment of their affective state. Participants are asked to respond quickly, almost instinctively, in order to make their evaluation as truthful as possible. This part is not timed: an indefinite time was allowed to provide the self-assessment to ensure that participants were not subjected to mindless pressure. Each experimental session lasts approximately 35 min.

### Ethical approval

The study was approved by the Universitá Campus Bio-Medico di Roma Ethics Committee (Ethical Approval N. 03/19 PAR ComEt CBM) and in accordance with the Declaration of Helsinki.

## Results

In this section, the performance results of the machine learning algorithms used to implement the affective state model are provided in terms of: (1) accuracies and F-score using all extracted features; (2) accuracies and F-score using the optimal feature set obtained with feature selection; (3) estimation time obtained by the implemented KNN, SVMc, SVMg and LDA. As mentioned in the previous section, four classification problems are discussed for each machine learning algorithm: two binary problems (high/low valence and high/low arousal) and two multi-class problems (4 classes and 5 classes).

**Figure 4:**
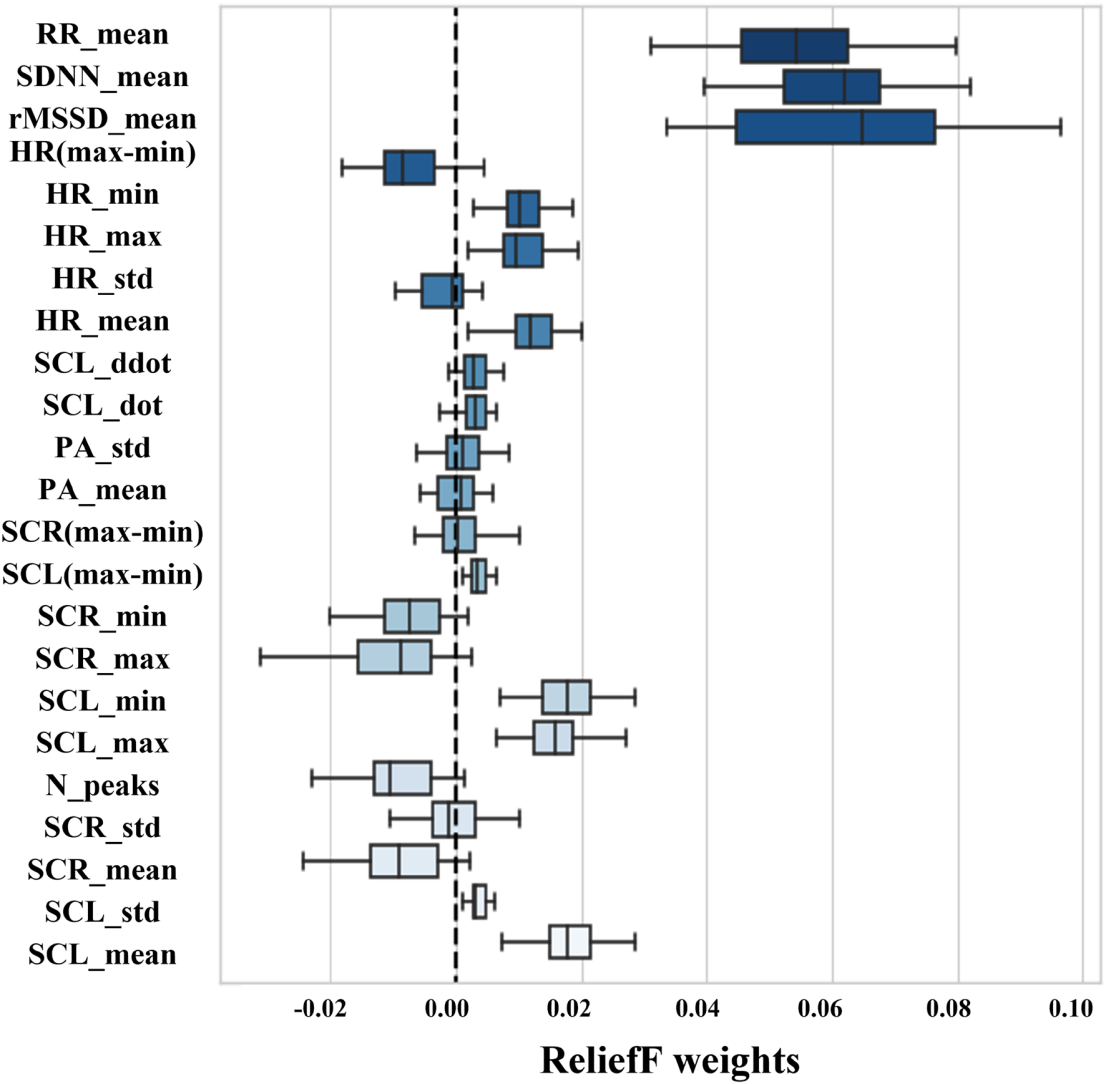
Boxplots of the weights for each feature provided by the use of the ReliefF algorithm for k = 5. The dashed line establishes the threshold for identifying the relevant features.

In Fig. 4, feature weights provided by ReliefF algorithm for each extracted feature are shown. A general consistency occurs among all the enrolled participants after ReliefF algorithm is applied: for k = 1, 3, 5, 10, the results of ReliefF algorithm implementation show that features with a positive weight are always the same across all the enrolled participants, regardless of the k parameter value selected: SCL_mean, SCL_std, SCL_max, SCL_min, SCL_dot, SCL_ddot, SCL_(max-min), HR_mean, HR_max, HR_min, sDNN_mean, rMSSD_mean, RR_mean. Feature selection reveals that the number of features with a median positive weight among all participants is 13 features from the original set of 23 features. During feature selection phase, the training dataset and the testing one remain separated. Some data are voluntarily left out to be used as new testing data never shown before.

**Figure 5:**
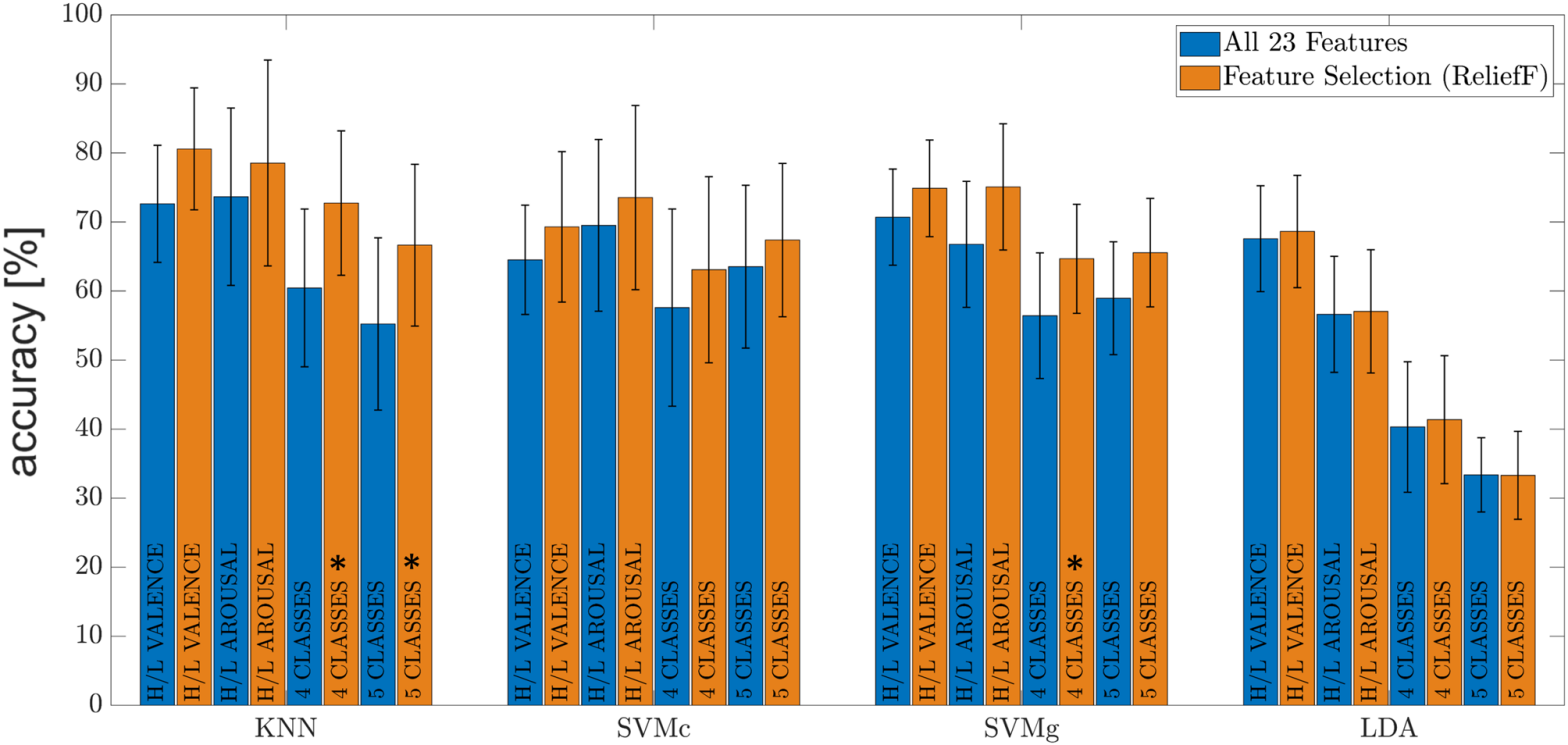
Accuracy of the machine learning algorithms estimation before (blue bars) and after (orange bars) feature selection using ReliefF algorithm. Stars highlight the models that significantly improve their performance after ReliefF.

In Fig. 5, the results of the average accuracy between all the 20 participants to the experiment obtained with machine learning algorithms KNN, SVMc, SVMg and LDA are reported for all four classification problems. In particular, the average accuracy achieved using all the 23 extracted features to train models is compared with one obtained using only the optimal feature set selected by ReliefF algorithm. As shown in the histogram, orange bars stand for the percentage increase in the average accuracy, for each machine learning algorithm, achieved considering only the optimal feature set. Blue bars indicate the average accuracy of machine learning algorithms achieved before the feature selection step. The black stars above the bars highlight the statistically significant improvements in the classification accuracy due to feature selection.

**Figure 6:**
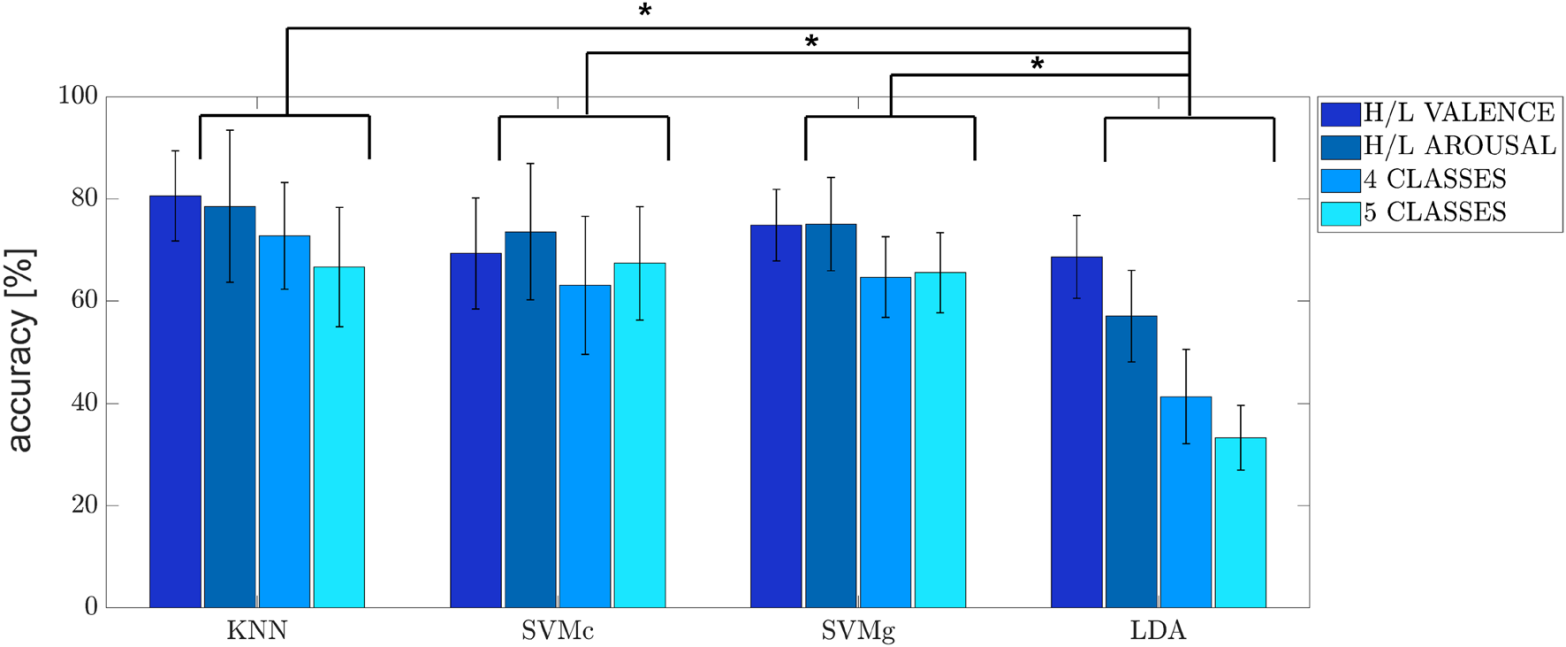
Machine learning algorithms accuracies of the four classification problems (i.e. H/L valence, H/L arousal, 4 classes and 5 classes) using the optimal feature set. Significant differences between machine learning algorithms are also highlighted with black stars.

The average accuracy values with standard deviations of all four classification problems using the optimal feature set are shown graphically in Fig. 6. Results of the statistical analysis are also reported: significant differences among machine learning algorithms are highlighted with black stars. Tables 2 and 3 report numerically the average accuracy values and F-score with standard deviations collected for all the participants for each machine learning algorithm using the optimal feature set.

**Table 2:** Accuracy computed for the machine learning algorithms on the test set using the optimal feature set.

Acc. (%)	H/L valence	H/L arousal	4 classes	5 classes
KNN	$80.5 \pm 12.6$	$78.5 \pm 21.2$	$72.7 \pm 14.9$	$66.5 \pm 16.7$
SVMc	$69.2 \pm 15.5$	$73.5 \pm 19.0$	$63.0 \pm 19.2$	$67.3 \pm 15.8$
SVMg	$74.8 \pm 10.0$	$75.0 \pm 13.0$	$64.6 \pm 11.2$	$65.5 \pm 11.2$
LDA	$68.6 \pm 11.6$	$57.0 \pm 12.7$	$41,3 \pm 13.2$	$33.2 \pm 9.0$

**Table 3:** F-score computed for the machine learning algorithms on the test set using the optimal feature set.

F-s (%)	H/L valence	H/L arousal	4 classes	5 classes
KNN	$80.2 \pm 15.1$	$77.8 \pm 15.5$	$73.7 \pm 9.0$	$66.5 \pm 9.0$
SVMc	$69.6 \pm 21.2$	$73.5 \pm 20.0$	$64.0 \pm 13.9$	$68.4 \pm 12.3$
SVMg	$76.2 \pm 18.4$	$75.1 \pm 19.9$	$65.8 \pm 11.4$	$66.8 \pm 12.8$
LDA	$69.2 \pm 18.1$	$59.4 \pm 17.2$	$43.2 \pm 11.1$	$34.1 \pm 10.0$

In addition to the accuracy analysis, the time required for each machine learning algorithm to return the prediction is also measured. Estimation times needed for real-time affective state estimation are averaged among all the participants and reported in Tables 4 and  5, using all 23 extracted features and using the optimal feature set respectively. These values take into account the time elapsed since the Physiological Monitoring Module receives new physiological raw data in input, pre-processes and filters them, extracts features and computes the affective state class.

**Figure 7:**
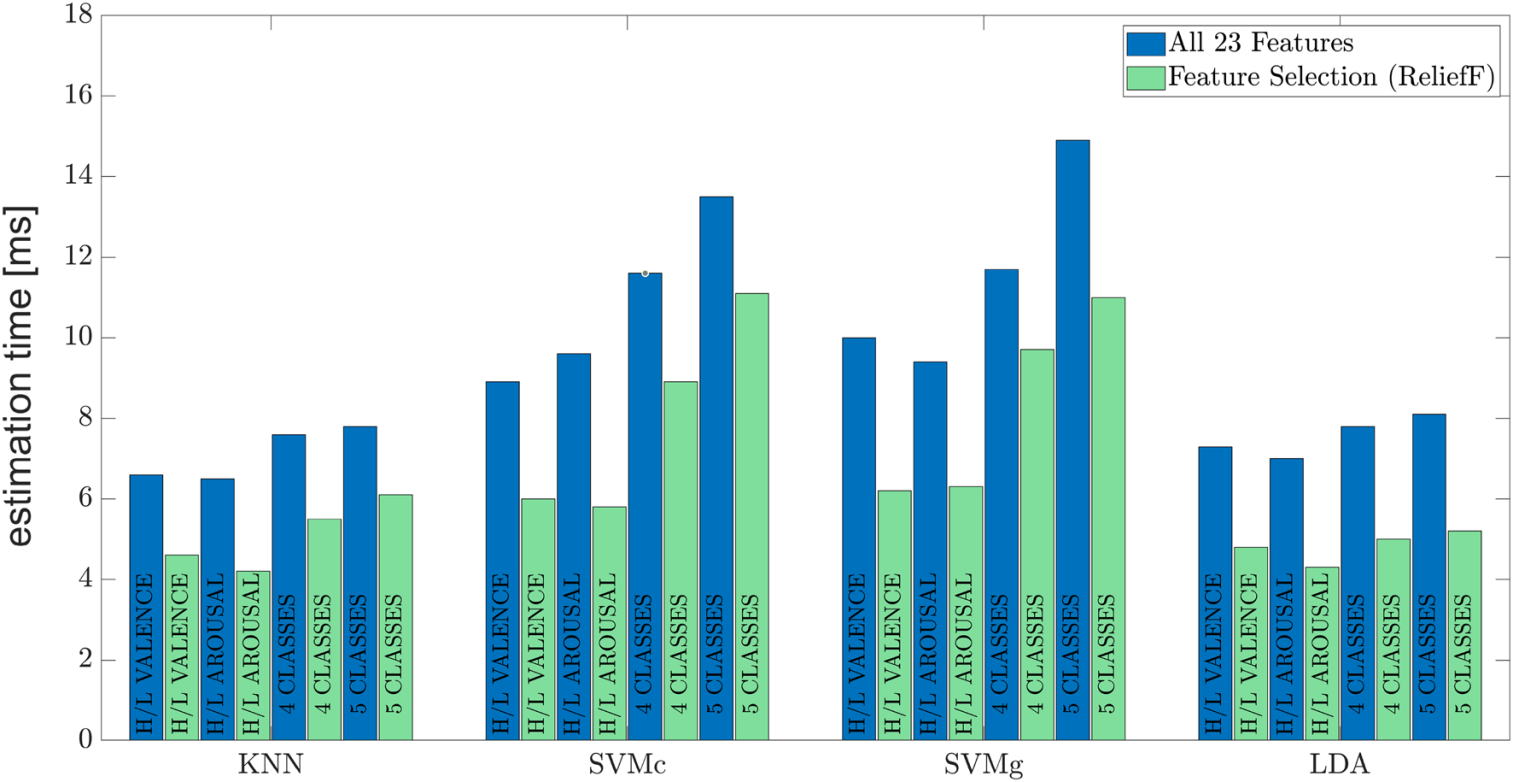
Mean estimation times using all 23 extracted features and the optimal feature set achieved with the ReliefF algorithm.

In Fig. 7, average estimation times in (ms) for each machine learning algorithm for both binary and multi-class classification problems are reported, considering feature selection (green bars) or not (blue bars). Lastly, a post-experiment analysis shows that the average response time of the participants for the self-assessment phase was 8.7 s (with a t_MAX_= 11.3 s and a t_min_= 4.5 s). This value is in agreement with the literature and with the aim of obtaining an instinctive self-assessment using a non-verbal method, such as SAM, without introducing a bias^49^.

Lastly, the impact of each sensor is assessed for the model that returned the highest mean classification accuray, i.e. KNN. In particular, the accuracy is computed on four different datasets taking into account all the computed features, only the features coming from the cardiorespiratory monitoring, only the features coming from the GSR and the optimal feature set obtained by applying the proposed feature selection approach. Table 6 reports the accuracy obtained for each tested dataset.

**Table 4:** Mean and standard deviation of the estimation times of each machine learning algorithm using all 23 extracted features.

time (ms)	H/L valence	H/L arousal	4 classes	5 classes
KNN	6.6 ± 3.5	6.5 ± 3.2	7.6 ± 3.9	7.8 ± 4.3
SVMc	8.9 ± 3.0	9.6 ± 2.7	11.6 ± 2.6	13.5 ± 2.3
SVMg	10.0 ± 2.9	9.4 ± 3.4	11.7 ± 2.6	14.9 ± 3.3
LDA	7.3 ± 3.1	7.0 ± 3.3	7.8 ± 3.5	8.1 ± 3.9

**Table 5:** Mean and standard deviation of the estimation time of each machine learning algorithm using the optimal feature set.

time (ms)	H/L valence	H/L arousal	4 classes	5 classes
KNN	4.6 ± 0.9	4.2 ± 0.5	5.5 ± 0.5	6.1 ± 0.7
SVMc	6.0 ± 0.3	5.8 ± 0.4	8.9 ± 1.2	11.1 ± 1.7
SVMg	6.2 ± 0.9	6.3 ± 0.5	9.7 ± 1.6	11.0 ± 1.2
LDA	4.8 ± 1.1	4.3 ± 0.5	5.0 ± 0.5	5.2 ± 0.5

**Table 6:** KNN Accuracy computed for the different classification problems varying the input dataset.

Accuracy (%)	H/L valence	H/L arousal	4 classes	5 classes
All 23 Features	$73.6\pm 12.1$	$73.7\pm 11.3$	$60.4\pm 9.9$	$55.2\pm 11.0$
Cardiorespiratory features	$73.2\pm 9.9$	$73.3\pm 10.4$	$68.7\pm 12.6$	$60.3\pm 12.4$
GSR features	$75.4\pm 6.2$	$75.2\pm 5.9$	$56.2\pm 14.2$	$51.6\pm 9.9$
Feature selection (relief)	$80.5 \pm 12.6$	$78.5 \pm 21.2$	$72.7 \pm 14.9$	$66.5 \pm 16.7$

## Discussion

Feature selection identifies which are the most relevant features for the classification of the affective state evoked by visual stimuli. The number of features with median positive weights is 13, starting from the initial set of 23 features. As shown in Fig. 4, ReliefF algorithm selects the same 13 features regardless of the considered k parameter value.

Among the initial 15 features extracted from the GSR signal, i.e the conductive changes of the skin due to the activity of the sweat glands, 7 features are selected by ReliefF algorithm as part of the final optimal feature set.

The remaining relevant features belonging to the optimal feature set derived from cardiac activity (5 features) and respiratory activity (1 feature). Significant weight values for the affective state estimation are assigned by the ReliefF algorithm to the features sDNN_mean, rMSSD_mean, RR_mean. SCL_mean resulted to be the most relevant feature from the galvanic skin response, as shown in Fig. 4. The features related to the trend of cardiac dynamics allow the calculation of quantitative and qualitative information describing the status of the Autonomic Nervous System. Heart Rate Variability is the oscillation of the heart rate over a series of consecutive heartbeats for a variable observation period. In the time domain, the most important parameters are the SDNN, i.e. the standard deviation of the “normal to normal” intervals, and the root mean square of successive differences between normal heartbeats (rMSSD). Both of these features, together with the RR_mean, which represents the number of respiratory acts, inspiration and expiration, per minute, have achieved a high positive weight value. These results show the importance of cardiac dynamics not only as regards the current heart rate, but also as regards the changes in heart rate over time. In other words, the heart rate variation over time is much more relevant for affective state estimation than the current heart rate. HRV is a good marker^50^, as well as respiratory rate, in identifying the complex physiological changes and processes of ANS that involve the human organism in correspondence to a certain affective state. Reducing the number of features by selecting fewer than 13 (e.g. 5 features) consequently decreases both the estimation time for classification (fewer features less computation time) and the accuracy rate (positively contributing features are removed). Since affective state changes have a period T on the order of seconds, the estimation time for classification on the order of milliseconds is already appropriate for real-time implementation. Therefore, there is no point to remove features with a positive weight in order to achieve faster times with a negative impact on accuracy: the bottleneck for fixing the optimal set at 13 features is dictated by the quest for the highest possible accuracy.

By examining the results in Fig. 5, the difference in the average accuracy for KNN, SVMc, SVMg and LDA using all 23 extracted features and using only the optimal feature set demonstrates how feature selection gives a strong contribution in improving algorithm accuracies. This is possible because feature selection enables to reduce the noise from the data and to select the most useful features to be employed during the training session. This greatly helps machine learning algorithm to become less overfit to the noises from the training data: as well as reducing computational complexity, a better generalization ability when tested with new unobserved data.

Analysing the variation in accuracy ranges, it is possible to observe a different impact of feature selection depending on the machine learning classifier used and the classification problem addressed: for the KNN, the use of the optimal feature set compared to the 23 features results in a significant increase in accuracy: + 8.0% (H/L valence), + 4.9% (H/L arousal), + 12.3% (4 classes), and + 11.4% (5 classes); for SVMc, the increase in accuracy ranges between + 3.9 and + 5.5%, while for SVMg, the increase in accuracy is between + 4.2 and + 8.3%. Accuracy improvements are more evident for KNN and SVM algorithms instead of LDA, where the increase is not very appreciable. An immediate visual analysis shows that the results obtained with the LDA algorithm are not particularly high in both circumstances, using all the features or using only the optimal feature set. This is attributable to the consideration that achieving good results with the LDA algorithm for these classification problems implies a linear relationship between the features extracted from physiological signals and the affective state evoked. However, as demonstrated by the results in terms of accuracy, nonlinear methods, such as KNN, achieve greater precision. For LDA, the accuracy does not benefit significantly from the use of the optimal feature set (0.4–1.1%).

Analysing the overall accuracy, high percentages of accuracy are hardly achievable since physiological signals are characterized by an intrinsic complexity of interpretation of their affective meaning and by high variability in typical values both in the same subject (intra-subject) and among several subjects (inter-subject)^51^. However, as shown in Fig. 5, good accuracy results are obtained: the highest accuracy result is achieved by the KNN for the classification problems H/L valence (80.5%), H/L arousal (78.5%), and 4 classes (72.7%), outperforming the other approaches. For the 5 classes problem, the highest accuracy is achieved by SVMc (67.3%), followed immediately by KNN (66.5%) and SVMg (65.5%). The accuracy for 5 classes is better for both SVMc and SVMg than for 4 classes, with an increase of + 4%. This may be due to the inclusion of an additional class (Neutral N), which changes the decision boundaries of the SVM method. These boundaries are rearranged and are capable of separating the data clusters, leading to an overall improvement in accuracy. In this manner, projected data close to the neutral condition are better discerned when the problem is split into 5 classes instead of 4.

Statistical analysis shows that the most significant improvement resulting from the introduction of the feature selection algorithm is manifested by the KNN algorithm. Multi-class problems benefit significantly from the feature selection step: $p=0.0005$ and $p=0.0036$ are the *p* values computed for the 4 and 5 classes problem respectively. A significant improvement is obtained also for the SVMg in 4 classes problem, with a *p* value of 0.0441. Statistically significant differences are not identified for both SVMc and LDA models (Fig. 5). Statistical analysis carried out on the accuracies returned by the implemented models showed that in H/L arousal and multi-class problems KNN and SVM outperform significantly the LDA classifiers (Fig. 6). In particular, the highest *p* value returned from the aforementioned comparisons is 0.006, obtained for SVMc-LDA.

In order to fully assess the quality of the predictive performance of machine learning algorithms in these kinds of classification problems, it is necessary not only to consider the accuracy values, but also to pay careful attention to precision and sensitivity. In classification problems, despite high accuracy values, precision and sensitivity could be inversely correlated giving rise to a classification that swings between excellent precision but poor sensitivity and vice versa. For this reason, it is important to obtain a fair compromise between these two parameters, called respectively precision and recall in the machine learning field. As shown in Table 3, the harmonic average between precision and recall (F-score values), reflects a balanced classification model in terms of precision and sensitivity, according to the accuracy values in Table 2.

As expected, the mean estimation time is reduced if the number of features to be taken into account is reduced from 23 to 13 since the number of operations that the model has to perform is lower. The estimation time using the optimal feature set is represented by the green histogram bars in Fig. 7. In detail, considering the average values of the estimation time: (1) KNN obtains the highest computation time reduction in the high/low arousal problem (2.3 ms) and the lowest in the 5 classes problem (1.7 ms); (2) SVMc achieves the highest computation time reduction in the high/low arousal problem (3.8 ms) and the lowest in the 5 classes problem (2.4 ms); (3) SVMg achieves the highest computation time reduction in the 5 classes problem (3.9 ms) and the lowest in the 4 classes problem (2.0 ms); (4) LDA achieves the highest computation time reduction in the 5 classes problem (2.9 ms) and the lowest in the high/low valence problem (2.5 ms). Furthermore, as shown in Table 4, high standard deviation values indicate high volatility: estimation times deviate significantly from the mean when all 23 features are used. Conversely, as shown in Table 5, the standard deviation values are lower, indicating low volatility: estimation times deviate less significantly from their average when the optimal feature set is used.

Lastly, an analysis is carried out to identify the specific contribution of the sensory systems to the classification accuracy. To this purpose, the KNN model was trained using different input datasets. The results shown in Table 6 show that in binary problems KNN achieved mean accuracies very similar in cases where all or only the features of a single sensor are exploited, whatever the BioHarness or Shimmer. Multiclass problems highlighted greater differences in performance. Specifically, cardiorespiratory features obtained higher accuracies with respect to the GSR ones. Anyhow, it resulted to be clear that the feature set obtained by applying the proposed feature selection method returned higher accuracy for all the classification problems faced in this paper. This highlights how multisensory integration allows one to address problems of affective state identification with greater accuracy.

Affective state changes are slow variations in time, in the order of seconds. Thus, it is verified that the estimation times are suitable and responsive for affective state evaluation. The main achievement is in reaching higher possible levels of accuracy using well-known machine learning classifiers since an erroneous estimation of affective state has a strongly negative impact on the person in human-centered technologies compared to a correct but slightly delayed estimation. Rather than an expected improvement over time, feature selection leads to a significant increase in accuracy to achieve a certain level of accuracy that can guarantee a reliable affective state outcome in estimation for the aforementioned application scenarios.

The performance of the model presented in this paper is dependent on the experimental conditions under which the datasets were acquired and the choice of features extracted from the raw data collected^52^. For the experimental conditions reported in this work, the feature set that emerged is the optimal one in terms of accuracy and computational burden. Using different experimental conditions and/or choosing different feature sets could alter the classification performance. The study is carried out by considering binary and multi-class classification problems: this methodological approach highlighted how, in an attempt to discriminate more and more affective states in the valence-arousal plane, the values of the classification accuracy inevitably decrease. The proposed affective state model is trained with labeled data from the experimental sessions and validated on a different testing set (i.e. made of non-labeled data). It aims at demonstrating the possible applicability of the proposed approach to real world applications, even in other settings where it is unlikely to dispose of the self-reported affective state. Depending on the application field where the affective estimation is to be addressed, the choice is boiled down either to a model with good accuracy that simplifies the affective state estimation at the binary case or to one that clearly distinguishes the affective state in the valence-arousal plane at the expense of accuracy itself. The right compromise between good accuracy and fine estimation of the affective state in the valence-arousal plane is reached by the KNN algorithm, which is also the most reactive in terms of estimation time for real-time human-centered applications and technologies.

## Conclusions

This paper has addressed the existing gaps in the literature to develop a reliable real-time affective state estimation model. An accurate analysis of the state of the art in this field revealed that neither a well-established optimal feature set nor a classification method effective in terms of accuracy and estimation time has still been determined. To overcome this, an optimal feature set has been identified from physiological signals recorded from galvanic skin response and from cardiac and respiratory activities. Furthermore, a comparative analysis of well-known machine learning algorithms, in both binary and multi-class classification problems, has been performed.

The identified optimal set of 13 features is the combination of the most relevant features relating to skin sweating and to cardiac and respiratory activity, and has proven, together with the machine learning algorithms selected in this study, to achieve the best performance in terms of accuracy and estimation time. The study revealed that the KNN algorithm is the most suitable machine learning algorithm for the implementation of a predictive model for affective state estimation given the classification accuracy and little estimation times, compatible with real-time applications ($\le 15$ ms). Anyhow, in real world applications, where it is unlikely to dispose of the self-reported affective state in real-time in any context, the performance of the affective state estimation model may be affected by environmental variability, the activity performed by the users and health condition.

Future work will be devoted to expanding the dataset, enrolling heterogeneous participants with a wider age range and investigating the contribution of additional sensors to detect other relevant biosignals: electroencephalography, body temperature or electromyography could provide interesting features and contribute to a more accurate and effective affective state identification. Moreover, facial expressions can be considered as additional inputs to support physiological signals monitoring with image-based emotion recognition.
